# Mulberrofuran G, a Mulberry Component, Prevents SARS-CoV-2 Infection by Blocking the Interaction between SARS-CoV-2 Spike Protein S1 Receptor-Binding Domain and Human Angiotensin-Converting Enzyme 2 Receptor

**DOI:** 10.3390/nu14194170

**Published:** 2022-10-07

**Authors:** Young Soo Kim, Buyun Kim, Eun-Bin Kwon, Hwan-Suck Chung, Jang-Gi Choi

**Affiliations:** Korean Medicine Application Center, Korea Institute of Oriental Medicine, Dong-gu, Daegu 41062, Korea

**Keywords:** coronavirus disease 2019, severe acute respiratory syndrome coronavirus 2, mulberry, mulberrofuran G, spike protein, ACE2 receptor

## Abstract

Despite the recent development of RNA replication-targeted COVID-19 drugs by global pharmaceutical companies, their prescription in clinical practice is limited by certain factors, including drug interaction, reproductive toxicity, and drug resistance. COVID-19 drugs with multiple targets for the SARS-CoV-2 life cycle may lead to a successful reduction in drug resistance as well as enhanced therapeutic efficacy, and natural products are a potential source of molecules with therapeutic effects against COVID-19. In this study, we investigated the inhibitory efficacy of mulberrofuran G (MG), a component of *Morus alba* L., also known as mulberry, which has been used as food and traditional medicine, on the binding of the spike S1 receptor-binding domain (RBD) protein to the angiotensin-converting enzyme 2 (ACE2) receptor, which is the initial stage of the SARS-CoV-2 infection. In competitive enzyme-linked immunosorbent assays, MG effectively blocked the spike S1 RBD: ACE2 receptor molecular binding, and investigations using the BLItz system and in silico modeling revealed that MG has high affinity for both proteins. Finally, we confirmed that MG inhibits the entry of SARS-CoV-2 spike pseudotyped virus and a clinical isolate of SARS-CoV-2 into cells, suggesting that MG might be a promising therapeutic candidate for preventing SARS-CoV-2 binding to the cell surface during early infection.

## 1. Introduction

Severe acute respiratory syndrome coronavirus 2 (SARS-CoV-2), which emerged in late 2019 and was later declared a pandemic [1], causes a severe respiratory syndrome known as coronavirus disease 2019 (COVID-19). During its life cycle, SARS-CoV-2 penetrates into human cells by attaching to the cell surface and subsequently producing viral genetic and structural materials before releasing the assembled and replicated viruses from the cell [2].

Small molecule-based drugs have been developed for COVID-19, including remdesivir (Gilead Sciences, Foster City, CA, USA), which has been approved by the U.S. Food and Drug Administration (FDA), and Paxlovid (Pfizer, New York, NY, USA) and molnupiravir (Merck & Co., Kenilworth, NJ, USA), which are authorized for emergency use, respectively; these drugs inhibit the RNA replication of SARS-CoV-2. Nevertheless, the emergence of a SARS-CoV-2 variant resistant to remdesivir, which suppresses RNA-dependent RNA polymerase (RdRp) activity, at approximately one year after the FDA approval implies that there is still a need to develop therapeutic agents for COVID-19 with mechanisms other than RdRp inhibition [3].

The spike protein, a crown-like spike glycoprotein protruding from the SARS-CoV-2 envelope, comprises two functional domains: the S1 receptor-binding domain (RBD), responsible for binding to an angiotensin-converting enzyme 2 (ACE2) receptor on the surface of human epithelial cells; and the S2 domain, responsible for cell fusion via processing by transmembrane serine protease 2 (TMPRSS2) [2]. Antibody-based drugs that inhibit the spike S1 RBD:ACE2 receptor interaction, which prevents SARS-CoV-2 from binding to cells in the initial phase of the viral infection process, have been developed by companies around the world: regdanvimab (Celltrion Healthcare, Inc., Incheon, Korea), casirivimab and imdevimab cocktail (Regeneron Pharmaceuticals, Inc., Tarrytown, NY, USA), and bamlanivimab and etesevimab cocktail (Eli Lilly and Company, Indianapolis, IN, USA). Despite the restriction of antispike S1 RBD antibody medications to intravenous or subcutaneous administration, spike S1 RBD:ACE2 inhibitors based on small molecules for oral administration will be able to provide better dose and efficacy convenience for patients. Thus, the exploration of ingredients from natural products may support the discovery of small molecule-based therapeutic candidates for COVID-19.

*Morus alba* L., also known as mulberry, has a long history of use as a food and traditional medicine. The nutritive value of its fruit is well established; it is used to make jams, marmalades, ice creams, vinegars, juices, and wines. Furthermore, the pharmacological value of its leaves, peels, and twigs include hypoglycemic, hypolipidemic, anti-inflammatory, antiatherogenic, treating fever, blood pressure lowering, and antioxidant effects [4,5,6,7,8,9,10]. Mulberry has been shown to have antiviral efficacy against herpes simplex virus-1 (HSV-1), influenza virus, and human norovirus (hCoV-229E) [11,12,13]. Through in silico modeling, Shakya et al. reported that several mulberry-derived compounds prevent TMPRSS2 from priming and cleaving the spike protein S2 domain [14].

Mulberrofuran G (MG), a bioactive phytochemical isolated from mulberry, has been studied for its antihepatitis B, antioxidant, neuroprotective, and antityrosinase activities [15,16,17,18]. Recently, it was investigated for anti-SARS-CoV-2 potential as an inhibitor of spike protein and 3C-like protease (3CL^pro^) inhibitor [19,20]. However, the effectiveness of MG against SARS-CoV-2 infection through inhibiting the spike S1 RBD:ACE2 receptor interaction has not yet been reported either in vitro or in vivo.

Therefore, in this study, we investigated the efficacy of MG, derived from mulberry, in the inhibition of the entry of SARS-CoV-2 into cells, through inhibiting the molecular interaction between the spike S1 RBD and the ACE2 receptor. We studied the effectiveness of MG in inhibiting the cellular infection of a clinical isolate of SARS-CoV-2 in vitro to determine its potential as a COVID-19 therapy.

## 2. Materials and Methods

### 2.1. Materials

MG was purchased from ChemFaces (Wuhan, China). The SARS-CoV-2 spike/ACE2 inhibitor screening assay kit, biotin-labeled recombinant protein ACE2 receptor, and spike protein S1 RBD (BPS Bioscience, San Diego, CA, USA) were procured for biolayer interferometry. HEK293T cells stably expressing human ACE2 and TMPRSS2 cells were obtained from GeneCopoeia (Rockville, MD, USA) and maintained in Dulbecco’s modified Eagle’s medium (Lonza, Walkersville, MD, USA) containing 10% fetal bovine serum (Biotechnics Research, Lake Forest, CA, USA) and 1% penicillin/streptomycin (Cellgro, Manassas, VA, USA) at 37 °C in a 5% CO_2_ incubator. Further, wild-type (WT) and mutant (D614G) SARS-CoV-2 spike pseudotyped viruses were purchased from GeneCopoeia (Rockville, MD, USA). Vero cells were purchased from American Type Culture Collection (Manassas, VA, USA). SARS-CoV-2 (βCoV/Korea/KCDC03/2020) was provided by Korea Centers for Disease Control and Prevention (Cheongju, Korea).

### 2.2. SARS-CoV-2 Spike/ACE2 Inhibitor Screening Assay

We assessed the binding of MG to the SARS-CoV-2 spike protein and the ACE2 receptor using the SARS-CoV-2 spike/ACE2 inhibitor screening assay kit (BPS Bioscience, San Diego, CA, USA) based on enzyme-linked immunosorbent assays (ELISAs). A 96-well plate was coated with 50 μL of 1 μg/mL SARS-CoV-2 spike protein in phosphate-buffered saline (PBS) overnight at 4 °C, washed with 100 μL of 1× immune buffer, blocked with 100 μL of 1× blocking buffer at room temperature for 1 h, and then washed again. Subsequently, 20 μL of 1× immune buffer was added to each well, followed by 10 μL of inhibitor solution containing the SARS-CoV-2 spike protein antibody (Active Motif, Carlsbad, CA, USA) or MG. After incubation at room temperature for 1 h with slow shaking, 20 μL of 2.5 μg/mL ACE2 inhibitor solution containing MG or SARS-CoV-2 spike antibody was added and incubated for 1 h at room temperature with slow shaking. After three washes in 100 μL of 1× immune buffer, 100 μL of 1× blocking buffer was added per well, incubated at room temperature for 10 min, and then washed again. Subsequently, 100 μL of anti-His-HRP was added to each well, and the plates were incubated for 1 h. Again, the plates were washed three times with 1× immune buffer, blocked with 100 μL of 1× blocking buffer per well at room temperature for 10 min, and washed again. Relative chemiluminescence was measured using a GloMax-multi microplate reader (Promega, Madison, WI, USA).

### 2.3. Kinetic Analysis of the Binding between MG and Spike Protein/ACE2 Receptor Based on Biolayer Interferometry

The binding affinities and kinetics of MG for the spike protein RBD and ACE2 receptor were evaluated using a BLItz system based on biolayer interferometry (Sartorius AG, Göttingen, Germany). After pre-equilibration of the streptavidin BLI sensor (Sartorius AG, Göttingen, Germany) in PBS buffer for 10 min, biotinylated spike protein S1 RBD and ACE2 receptor were fully loaded onto the BLI sensors by soaking the sensor in 4 μL of 50 μg/mL spike protein RBD and ACE2 receptor solution. The MG solutions for kinetic analysis were prepared by diluting the stock solution in PBS with 1% dimethyl sulfoxide (DMSO) to a final volume of 0, 50, 100, 200, and 400 nM.

The protein-immobilized sensor was first equilibrated in PBS buffer containing 1% DMSO for 10 s. Next, 4 μL of MG was associated with the equilibrated protein-immobilized sensor for 10 s, followed by dissociation in PBS containing 1% DMSO for 10 s. The degree of MG binding was represented as a shift in wavelength (nm) between the bound and reference signals in the biosensor. The kinetic constants were calculated using BLItz Pro by fitting the association and dissociation data to a 1:1 model. The equilibrium dissociation constant, *K*_D_, was calculated as the dissociation constant divided by the association constant (*k*_d_/*k*_a_).

### 2.4. In Silico Docking Simulation and Pharmacophore Analysis

The structures of the SARS-CoV-2 spike protein and ACE2 receptor were retrieved from the Protein Data Bank (www.rcsb.org, PDB code: 6M0J) as receptors for docking simulations; MG (PubChem ID: 196583) was used as a ligand. MG was docked in a space with dimensions of 25 × 40 × 30 units with the SARS-CoV-2 spike protein and ACE2 receptor facing each other. Interactions were determined using AutoDock Vina integrated with UCSF Chimera 1.15. The binding affinity between MG and the spike protein/ACE2 receptor complex was presented as the lowest energy score in the docking simulation. The molecular interactions between MG and the spike protein/ACE2 receptor were analyzed and visualized as receptor–ligand interactions on a 2D diagram in BIOVIA Discovery Studio Visualizer v20.1.0.19295 (Dassault Systèmes, Vélizy-Villacoublay, France).

### 2.5. Cell Viability Assay

Cell viability was determined using the 3-[4,5-dimethylthiazol-2-yl]-2,5 diphenyl tetrazolium bromide (MTT) assay. HEK293T cells were seeded into 96-well plates at 5 × 10^4^ cells/well, and MG was added to the wells at concentrations of 0–50 μM. MTT solution was added to each well after 24 h, and the cells were incubated for a further 30 min [21,22]. Subsequently, 1 mL dimethyl sulfoxide was added before the absorbance at 540 nm was measured using an Epoch Microplate Reader (BioTek, Winooski, VT, USA). The data were presented as the mean ± SEM of four independent experiments.

### 2.6. SARS-CoV-2 Lentiviral Pseudovirus Infection Assay

The green fluorescent protein (GFP) spike (SARS-CoV-2) pseudotyped lentivirus infection test was performed in HEK293T cells. Briefly, HEK293T cells stably expressing human ACE2 and TMPRSS2 were cultured in 96-well plates (5 × 10^4^ cells/well) for 18 h. Next, WT and mutant (D614G) SARS-CoV-2 spike pseudovirus (at a final concentration of 1 × 10^4^ TU/mL to each well) was incubated with 2 μM MG or anti-SARS-CoV-2 spike antibody at 37 °C for 1 h. These mixtures were then added to HEK293T cells. The infection levels were determined at 3 days post infection. The reduction in the SARS-CoV-2 spike pseudovirus infection was estimated by measuring GFP expression using flow cytometry and fluorescence microscopy (Olympus, Tokyo, Japan).

### 2.7. Hoechst Staining and Immunofluorescence Assay

Vero cells were cultured for 24 h after inoculation at 1.2 × 10^4^ cells per well in 384-well plates, and treated with MG in 2-fold serial dilutions for 1 h. Then, cells were infected with 0.0125 MOI of SARS-CoV-2 and incubated at 37 °C for 24 h. After fixation with 4% paraformaldehyde, the permeabilized cells were stained with anti-SARS-CoV-2 nucleocapsid (N) primary antibody, Alexa Fluor 488-conjugated goat anti-rabbit IgG secondary antibody, and Hoechst 33342. Operetta (Perkin Elmer, Waltham, MA, USA) was used to acquire fluorescent images of infected cells, which were then analyzed using Columbus software (Perkin Elmer).

### 2.8. Statistical Analysis

The data presented as the mean ± standard error of the mean were analyzed using GraphPad PRISM software (v5.02; GraphPad USA).

## 3. Results

### 3.1. The Blockade of Spike S1 RBD:ACE2 Receptor Interaction by MG

**Figure 1:**
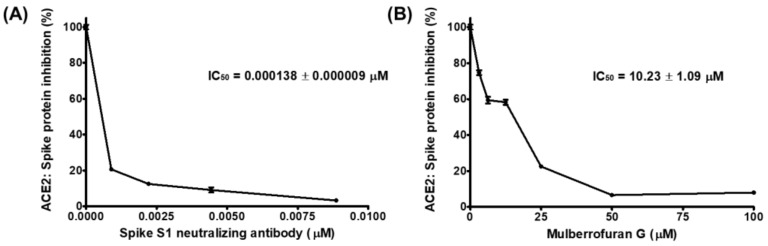
Inhibitory effect of mulberrofuran G (MG) on the binding of spike protein S1 receptor-binding domain (RBD) to the human angiotensin-converting enzyme 2 (ACE2) receptor. Spike protein coated on a 96-well plate interacted with a preincubated mixture of the ACE2 receptor and (**A**) antibody to the spike protein as the positive control and (**B**) 0, 3.125, 6.25, 12.5, 25, 50, or 100 μM MG. The inhibition of the binding of the spike S1 RBD to the ACE2 receptor by MG was evaluated by measuring chemiluminescence.

We investigated the potential of MG to inhibit SARS-CoV-2 entry into cells by using competitive ELISA to assess the blockade of molecular binding between the spike and ACE2 proteins (Figure 1). The positive control, spike S1 neutralizing antibody, blocked the binding of treated ACE2 protein to the spike S1 RBD coated on the plate with an IC_50_ value of 19.45 ng/mL. At concentrations below 50 μM, mulberrofuran G also effectively reduced the interaction between spike S1 RBD and ACE2; the IC_50_ value was 10.23 μM, which indicated that mulberrofuran G could hamper SARS-CoV-2 entry into the cells by interfering with the spike:ACE2 interaction.

### 3.2. Kinetic Analysis of MG:spike S1 RBD/ACE2 Receptor Interaction

**Figure 2:**
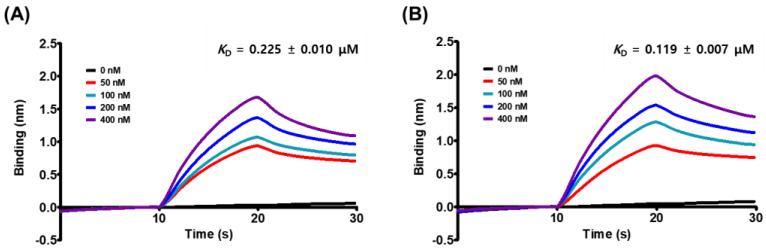
The global kinetic analysis of mulberrofuran G (MG) binding to biotinylated (**A**) ACE2 receptor and (**B**) spike S1 RBD immobilized on a streptavidin-coated sensor. The kinetics of MG for the spike S1 or ACE2 receptor were measured from the association of 0, 50, 100, 200, and 400 nM of MG in PBS containing 1% DMSO with immobilized spike S1 or ACE2 receptor and the subsequent dissociation in PBS containing 1% DMSO. The degree of MG binding was represented as a shift in wavelength (nm) between the bound and reference signals in the biosensor.

We evaluated the affinity of MG to the spike S1 RBD and the ACE2 receptor to determine the target specificity of MG using a kinetic model based on the biolayer interferometry-based BLItz system. MG strongly bound to both proteins, with the spike S1 RBD exhibiting a slightly higher affinity than the ACE2 receptor: the equilibrium dissociation constant (*K*_D_) of MG for the spike S1 RBD was 0.119 μM and that for the ACE2 receptor was 0.225 μM (Figure 2 and Table 1). Further kinetic analysis revealed that MG was more likely to associate as well as dissociate from the spike S1 RBD relative to the ACE2 receptor (Table 1), resulting in the slight discrepancy in the affinity of MG to each protein. However, this strong binding of MG to both proteins suggested that MG can prevent SARS-CoV-2 from attaching to human epithelial cells by targeting both proteins.

### 3.3. Molecular Docking Simulation and Pharmacophore Analysis of MG with Spike Protein and ACE2 Receptor

**Figure 3:**
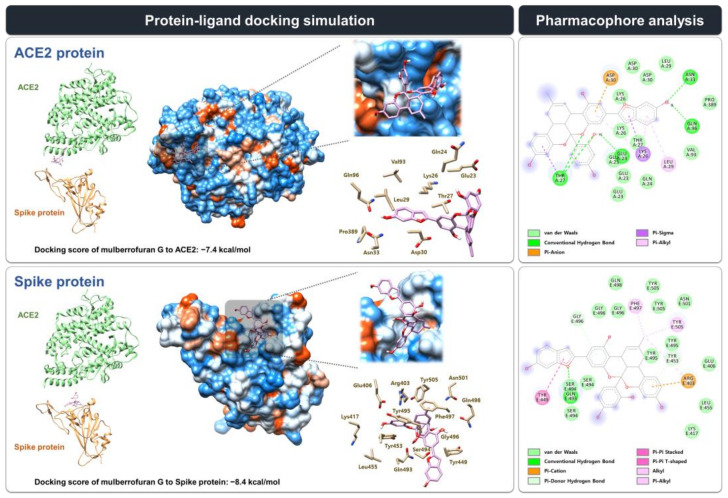
Protein–ligand docking simulation between mulberrofuran G (MG) and the ACE2 receptor/spike protein. MG docking onto the SARS-CoV-2 spike protein and ACE2 receptor (PDB code 6M0J) was simulated using AutoDock Vina. The pharmacophore analysis of MG with the spike protein or hACE2 receptor was performed using BIOVIA Discovery Studio Visualizer.

We estimated the binding mode and interaction of MG with the spike protein and the ACE2 receptor at the interface where two proteins face each other and interact using in silico modeling (Figure 3). The docking simulation showed that MG might bind more stably to the spike protein than to the ACE2 receptor, with a lower docking score (∆G) of −8.4 kcal/mol compared with −7.4 kcal/mol, respectively, which was consistent with the biolayer interferometry analysis.

Receptor–ligand interaction research into the MG docking modes indicated that MG might form 26 and 23 interactions, respectively, with the amino acid residues located at the interface where the spike protein and ACE2 receptor face each other: spike protein, 14 van der Waals interactions (Glu406, Lys417, Leu455, Ser494, Tyr495, Gly496, Gln498, Asn501, and Tyr505), 1 conventional hydrogen bond (Gln493), 2 pi–donor hydrogen bonds (Tyr453 and Gly496), 1 pi–pi stacking interaction (Tyr449), 1 pi–pi T-shaped interaction (Tyr449), 3 alkyl interaction (Arg403, Phe497, and Tyr505), 3 pi–alkyl interaction (Arg403, Phe497, and Tyr505), and 1 pi–cation interaction (Arg403); ACE2 receptor, 12 van der Waals interactions (Glu23, Gln24, Lys26, Thr27, Leu29, Asp30, Val93, and Pro389), 5 conventional hydrogen bonds (Glu23, Thr27, Asn33, and Gln96), 2 pi–sigma interactions (Lys26 and Thr27), 3 pi–alkyl interaction (Lys26 and Leu29), and 1 pi–anion interaction (Asp30) (Figure 3).

### 3.4. MG Inhibits SARS-CoV-2 Lentiviral Pseudovirus Infection in HEK293T Cells Overexpressing ACE2/TMPRSS2

**Figure 4:**
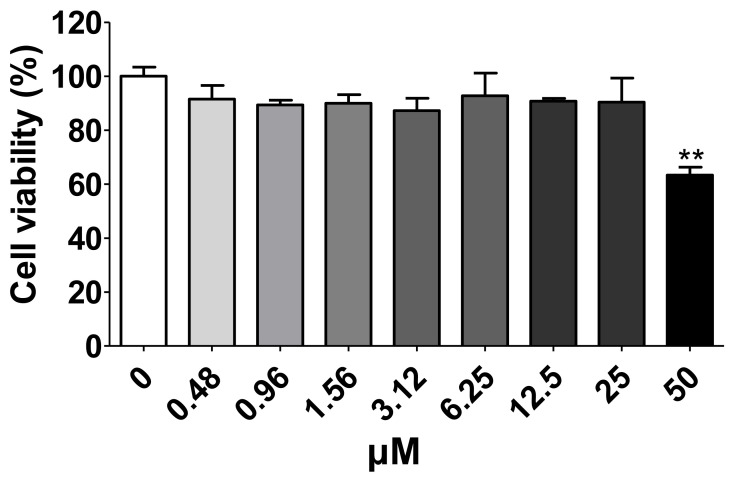
Determination of the cytotoxic effects of different concentrations of mulberrofuran G (MG) in HEK293T cells stably expressing human ACE2 and TMPRSS2. Cell viability after 24 h was determined by MTT (3-(4,5-dimethylthiazol-2-yl)-2,5 diphenyl tetrazolium bromide) assay. Bar graph (mean ± SEM) statistics were determined by three experimental datasets using one-way ANOVA with Tukey’s posthoc test; ** *p* < 0.01, compared with untreated sample (0 μM MG).

The cytotoxic effects of MG were investigated by incubating HEK293T cells expressing human ACE2 and TMPRSS2 with 0–50 μM MG for 24 h. However, MG did not exhibit cytotoxic effects at 25 μM in HEK293T cells (Figure 4). Therefore, subsequent experiments were conducted with MG concentrations below 25 μM.

**Figure 5:**
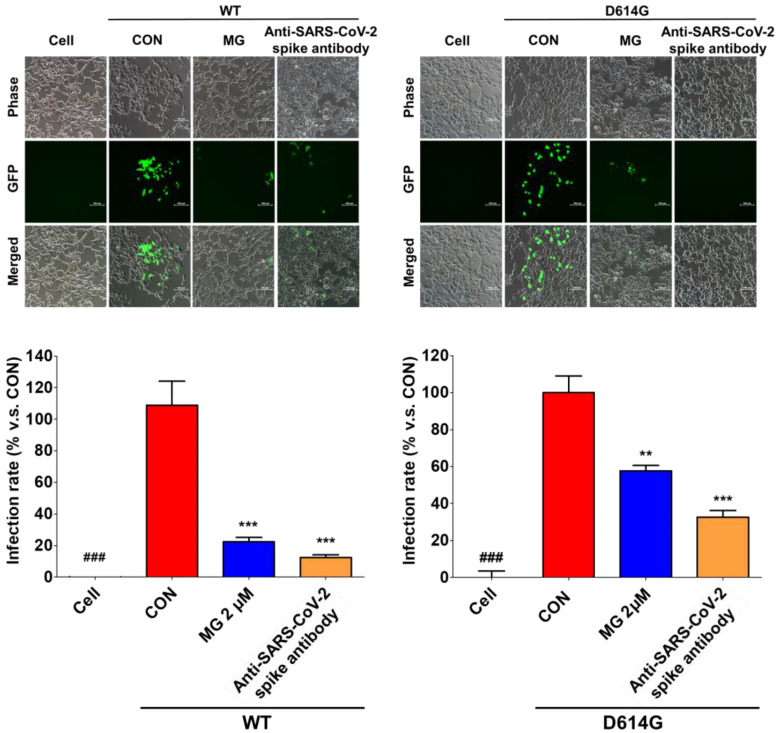
Mulberrofuran G (MG) inhibits SARS-CoV-2 lentiviral pseudovirus infection in HEK293T cells stably expressing human ACE2 and TMPRSS2 cells. HEK293T cells were cultured in 96-well plates (5 × 10^4^ cells/well) for 18 h. Next, wild-type (WT) or mutant (D614G) SARS-CoV-2 spike pseudovirus (at a final concentration of 1 × 10^4^ TU/mL to each well) was incubated with MG (2 μM) or anti-SARS-CoV-2 spike antibody, and the mixtures were incubated at 37 °C for 1 h and then added to HEK293T cells. Green fluorescent protein (GFP) expression levels using flow cytometry were assessed at 72 h after viral infection. The inhibitory effect on SARS-CoV-2 spike pseudovirus infection was determined by measuring GFP expression using flow cytometry under a fluorescence microscope. Bar graph (mean ± SEM) statistics were determined by three experimental datasets using one-way ANOVA with Tukey’s posthoc test, *** *p* < 0.001; ** *p* < 0.01, compared with the control (CON, no MG treatment) samples; ^###^ *p* < 0.001, compared with the cell-only sample.

We evaluated the ability of MG to prevent SARS-CoV-2 infection by interfering with SARS-CoV-2 attachment to the cellular surface. After treatment with 2 μM MG, ACE2/TMPRSS2-overexpressing HEK293T cells were infected with SARS-CoV-2 spike pseudotyped virus, a lentivirus in which the vesicular stomatitis G (VSV-G) envelope glycoprotein was replaced with WT and mutated (D614G) forms of the SARS-CoV-2 spike protein. We observed that MG-treated HEK293T cells had significantly lower GFP expression than the untreated cells, which showed high GFP expression levels upon infection with WT and mutant (D614G) SARS-CoV-2 lentiviral pseudovirus at 72 h (Figure 5). Additionally, flow cytometry using a fluorescence detection assay showed that MG effectively inhibited SARS-CoV-2 spike pseudotyped viruses in ACE2/TMPRSS2-overexpressing HEK293T cells (Figure 5). These results indicated that MG-treated cells have significantly lower SARS-CoV-2 spike pseudotyped virus infection compared with untreated cells.

### 3.5. MG Inhibits the Infection of a Clinical Isolate SARS-CoV-2 in Vero Cells

**Figure 6:**
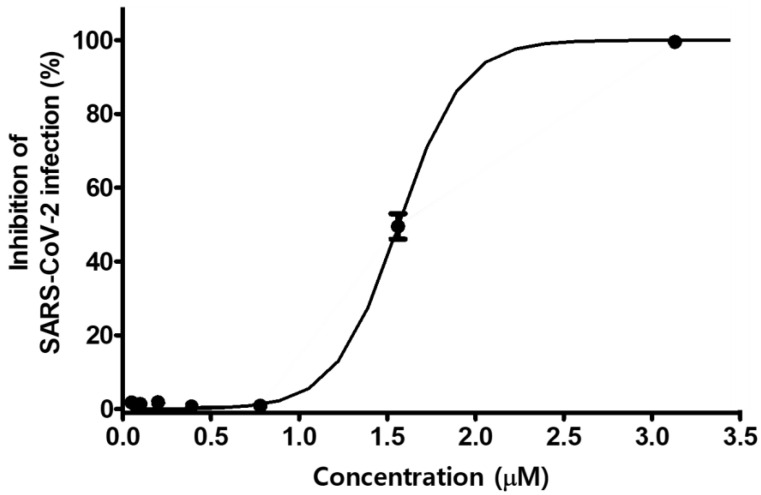
Mulberrofuran G (MG) inhibits the infection of a clinical isolate of SARS-CoV-2 in Vero cells. Vero cells were cultured in 384-well plates (1.2 × 10^4^ cells/well) for 24 h. Cells treated with serial dilutions of MG were infected with SARS-CoV-2 (MOI 0.0125) and incubated at 37 °C for 24 h. The cells were then stained with anti-SARS-CoV-2 nucleocapsid (N) primary antibody, Alexa Fluor 488-conjugated goat antirabbit IgG secondary antibody, and Hoechst 33342.

We further evaluated the antiviral efficacy of MG against SARS-CoV-2 isolate (βCoV/Korea/KCDC03/2020) in Vero cells (ATCC-CCL81) and African green monkey kidney epithelial cells expressing a high level of the ACE2 receptor. Cells were infected with 0.0125 MOI of SARS-CoV-2 in the presence of MG at various doses. The inhibitory effect of MG on SARS-CoV-2 infection was determined by Hoechst staining and immunofluorescence assay using SARS-CoV-2 nucleocapsid protein antibody and Alexa Fluor 488-conjugated IgG. SARS-CoV-2 infection was significantly inhibited by treatment with MG at concentrations over 0.78 μM, whereas no inhibition of SARS-CoV-2 infection was observed at concentrations less than 0.78 μM MG. The 50% inhibitory concentration (IC_50_) of MG for SARS-CoV-2 infection was 1.55 μM (Figure 6). This showed that MG successfully prevents SARS-CoV-2 infection in a clinical isolate.

## 4. Discussion

Natural products have long been used as folk remedies against various diseases, and their constituent components have proven beneficial in providing backbones for various drug development processes. Many natural products have been investigated using virtual screening based on molecular docking simulation and an in vitro inhibitory assay in Vero cells highly expressing the ACE2 receptor for the development of a COVID-19 treatment [23,24,25].

To date, small molecule-based drugs for COVID-19 have been developed, such as remdesivir, Paxlovid, and molnupiravir, which inhibit SARS-CoV-2 RNA replication. However, molnupiravir, a nucleoside analog that can interfere with RNA replication, should be administered with caution in pregnant and lactating patients due to concerns regarding reproductive toxicity. In addition, inhibitors of viral RNA replication have led to acquired drug resistance due to the mutations frequently caused by ongoing viral replication and persistent drug exposure. Indeed, remdesivir-resistant SARS-CoV-2 was even found in November 2021, approximately one year after it received FDA approval as a therapeutic drug for COVID-19 [3].

**Figure 7:**
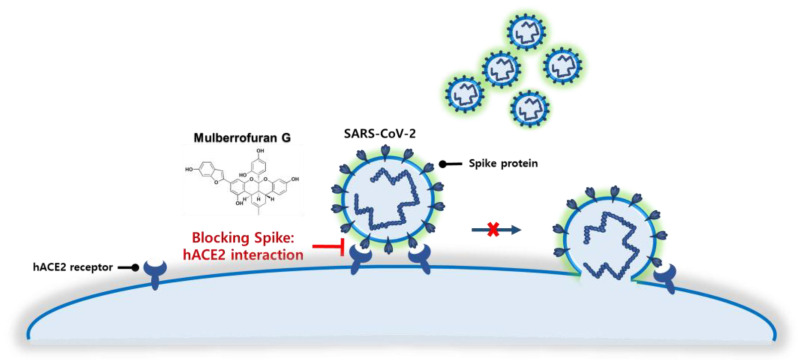
Schematic of the blockade of the SARS-CoV-2 spike S1 receptor-binding domain:angiotensin-converting enzyme 2 receptor interaction by mulberrofuran G.

As SARS-CoV-2 infection begins with the binding of the spike protein to the ACE2 receptor in human epithelial cells, which results in the SARS-CoV-2 entry into the cells, inhibiting the spike S1 RBD:ACE2 receptor interaction is a promising target for blocking SARS-CoV-2 infection at the source. Thus, several global pharmaceutical companies have developed and received FDA approval for antibody therapies that target the spike protein: Celltrion Healthcare, Inc.; Regeneron Pharmaceuticals, Inc.; and Eli Lilly and Company. However, antibody therapies targeting the SARS-CoV-2 spike protein have two drawbacks: production, which requires cell-based expression, and administration, which necessitates long-term intravenous injection. Therefore, small molecule-based therapeutics that block the spike S1 RBD:ACE2 receptor interaction will provide cost effective and easy-to-administer therapy for patients with COVID-19. In this work, we investigated the prevention of SARS-CoV-2 infection by blocking the spike S1 RBD:ACE2 receptor interaction with MG, a component of mulberry (Figure 7).

A competitive ELISA experiment, in which the spike S1 RBD was treated with the ACE2 receptor in a microplate, was used to assess the effectiveness of MG in inhibiting the spike S1 RBD:ACE2 receptor interaction. MG, similar to the spike S1 RBD-neutralizing antibody, effectively inhibited the binding of the spike S1 RBD to the ACE2 receptor in a dose-dependent manner with an IC_50_ value of 10.23 μM, indicating that MG has the potential to inhibit SARS-CoV-2 entry into cells by preventing SARS-CoV-2 from binding to the epithelial cell surface (Figure 1). The strong affinity of MG for both the spike S1 RBD and the ACE2 receptor, as assessed by the equilibrium dissociation constant (*K*_D_) in the biolayer interferometry study, substantiated its blocking effectiveness. MG was highly strongly bound to both target proteins, with *K*_D_ values of 0.119 μM (spike S1 RBD) and 0.225 μM (ACE2 receptor) (Figure 2 and Table 1). This result indicates that MG can prevent SARS-CoV-2 infection by concurrently targeting both proteins in the human body, despite the slightly higher affinity of MG to the spike S1 RBD than the ACE2 receptor, which was supported by the estimation of MG’s binding affinity (binding score) to each protein based on in silico docking simulation (Figure 3).

To date, although virtual screening studies have suggested that MG displayed anti-COVID-19 potential that inhibits spike protein interaction [19] and 3CL^pro^ activity [20], which are necessary for viral entry and replication, respectively, further in-depth investigation of the spike S1 RBD:MG interaction is required. We estimated the receptor–ligand interactions between MG and the spike S1 RBD/ACE2 receptor using pharmacophore analysis, resulting in 26 and 23 interactions with the spike protein and ACE2 receptor, respectively. Lan et al. employed X-ray crystallography to investigate the interaction between the spike protein and the ACE2 acceptor (PDB code: 6M0J) [26], indicating that various amino acids of the SARS-CoV spike protein, such as Tyr442, Leu472, Asn479, and Thr487, are critical for binding to the ACE2 receptor, which corresponds to Leu455, Phe486, Gln493, and Asn501 of SARS-CoV-2. The docking simulation in this study suggested that MG interferes with the interactions between the spike S1 RBD and the ACE2 receptor amino acids Asp30, Lys31, His34, Glu35, Tyr41, Lys353, Gly354, and Asp355 by forming van der Waals and hydrogen bonds with amino acids of the SARS-CoV-2 spike protein, including Leu455, Gln493, and Asn501. In the spike protein:MG binding mode, MG bound to the SARS-CoV-2 spike protein tyrosines (Tyr449 and Tyr505), which have multiple polar hydroxyl groups that are implicated in hydrogen-bonding with the ACE2 receptor, via van der Waals, pi–pi, alkyl, and pi–alkyl interactions. Lan et al. found that unlike SARS-CoV, the SARS-CoV-2 spike protein Lys417, which is located outside the S1RBD, only formed a unique salt bridge with the ACE2 receptor Asp30, which might explain the difference in the binding affinity between SARS-CoV-2 and SARS-CoV to the ACE2 receptor. Thus, we speculated that the interaction between MG and the SARS-CoV-2 spike protein Lys417 interferes with the formation of this salt bridge via van der Waals interaction. Furthermore, pharmacophore analysis suggested that MG might bind to Asp30 and Gln24 of the ACE2 receptor, which interact with the SARS-CoV-2 spike protein Leu455, Lys417, and Phe486, respectively, through van der Waals and pi–anion interactions.

As SARS-CoV-2 is a biosafety level (BSL)-3 virus, spike:ACE2 binding-mediated SARS-CoV-2 infection can be easily assessed in BSL-2 with a simplified assay based on a pseudotyped lentivirus particle in which the lentiviral VSV-G envelope protein has been substituted with the SARS-CoV-2 spike protein. The SARS-CoV-2 spike pseudotyped virus can enter the cells by attaching to the surface of ACE2/TMPRSS2-overexpressing HEK293T cells, and its infectivity can be assessed by GFP fluorescence. MG decreased SARS-CoV-2 spike pseudotyped virus infection in the ACE2/TMPRSS2-overexpressing HEK293T cells, as confirmed by decreased GFP fluorescence (Figure 5). Our result demonstrated the mechanism of action of MG in vitro, namely, that MG prevents SARS-CoV-2 infection by blocking the spike:ACE2 interaction.

Moreover, MG also effectively prevented the clinical isolate SARS-CoV-2 from infecting Vero cells (Figure 6). By combining the prior molecular binding studies and cell infection results, we concluded that MG blocked SARS-CoV-2 from entering epithelial cells as a mode of action that prevents the spike S1 RBD:ACE2 receptor interaction. However, the cytotoxicity of MG in Vero cells necessitates careful dosage control in COVID-19 treatment.

In this study, we confirmed the in vitro potential of MG, a component of mulberry, to inhibit cell entry by SARS-CoV-2 by blocking the spike S1 RBD:ACE2 receptor interaction. This suggests that MG, as well as mulberry, may be effective as a COVID-19 therapy and functional food to prevent SARS-CoV-2 infection. Previous studies have also reported on small compounds that inhibit SARS-CoV-2 entry. After screening a natural and FDA-approved compound libraries using homogeneous time resolved fluorescence, Li et al. found that TS-984 (9-methoxycanthin-6-one) suppresses the spike:ACE2 interaction via pseudovirus neutralization assay on ACE2-overexpressing 293T cells, suggesting that it may have potential as an anti-COVID-19 drug [27]. Wang et al. also confirmed the binding affinity of H69C2, which was virtually screened from a natural product database, to the RBD domain of SARS-CoV-2 spike protein using native mass spectrometry and surface plasmon resonance for binding: *K*_D_ value of 0.0947 µM. They verified that H69C2 effectively prevented SARS-CoV-2 infection in Vero E6 cells, with an IC_50_ of 85.75 µM [28]. In addition, Puhl et al. found the therapeutic candidate carvedilol as a SARS-CoV-2 spike inhibitor by using computational docking simulations of an FDA-approved drug library [29]. Carvedilol showed strong binding affinity with a *K*_D_ value of 0.364 μM and high antiviral effectiveness with an IC_50_ for SARS-CoV-2 infection of 7.57 μM in Calu-3 cells, indicating its potential as a spike RBD inhibitor [29]. However, the binding affinity and antiviral effectiveness of these SARS-CoV-2 entry inhibitors may be affected by various factors, including the measurement protocol, MOI, cell types, and the presence of additional anti-COVID-19 mechanisms. Furthermore, an in vivo study of SARS-CoV-2 infecting a human ACE2 transgenic mouse model in an animal biosafety level 3 (ABSL-3) laboratory will be able to confirm the potential of MG to act as a cell entry inhibitor as well as confirm the use of mulberry for the treatment of COVID-19 and, potentially, other diseases.

## Figures and Tables

**Table 1:** The binding kinetics of mulberrofuran G to spike S1 RBD and ACE2 receptor.

	*K*_D_ (μM)	*k*_a_ (μM^−1^ s^−1^)	*k*_d_ (s^−1^)	*R* ^2^
ACE2 receptor	2.25 × 10^−1^	2.43 × 10^−1^	5.46 × 10^−2^	0.9943
Spike S1 RBD	1.19 × 10^−1^	3.90 × 10^−1^	4.63 × 10^−2^	0.9958

## Data Availability

The data that support the findings of this study are available from the corresponding author upon reasonable request.
